# Dynamic variation in a combined inflammation–tumor marker index during neoadjuvant chemotherapy and its value for organ-preservation decisions in gastric cancer

**DOI:** 10.1515/biol-2025-1238

**Published:** 2026-01-23

**Authors:** Ke Liu, Haiquan Qian, Shensi Chen, Shengjun Zhang, Wei Zhao

**Affiliations:** Department of Gastrointestinal Surgery, The General Hospital of Ningxia Medical University, Yinchuan, 750001, China; Department of Nutrition, The General Hospital of Ningxia Medical University, Yinchuan, 750001, China

**Keywords:** gastric cancer, neoadjuvant chemotherapy, combined inflammation–tumor marker index, pathologic complete response, organ preservation

## Abstract

We evaluated whether on-treatment change in a Combined Inflammation–Tumor Marker Index (ΔCITI) signals chemosensitivity during neoadjuvant chemotherapy (NAC) for gastric cancer and can support selective organ preservation. Of 179 patients, 158 (88.3 %) completed planned NAC, 164 (91.6 %) underwent resection, with pathology evaluable in 163. *A* ≥ 1-point ΔCITI decline occurred in 53.1 % (95/179) as cohort means fell from 4.3 to 3.2. Among resected patients (evaluable *n* = 163), favorable ΔCITI was associated with higher pCR (22.5 % vs 9.8 %), R0 resection was achieved in 148/163 (90.8 %). In multivariable Cox models adjusting for regimen (FLOT vs SOX), baseline CITI, and clinicopathologic factors, favorable *Δ*CITI predicted improved overall survival (adjusted HR 0.62, 95 % CI 0.43–0.91) and showed a directionally favorable disease-free survival (adjusted HR 0.76, 95 % CI 0.55–1.06). A multidisciplinary pathway identified 15/179 (8.4 %) candidates for organ preservation, 11 proceeded, and 2/11 (18.2 %) experienced local regrowth, both salvaged. Given the small size and limited follow-up of this subgroup, these findings are exploratory. ΔCITI ≥ 1 offers a simple, dynamic indicator that complements imaging and endoscopy/biopsy for NAC response assessment and cautious MDT-guided de-escalation. Prospective validation and head-to-head comparisons with established indices are warranted.

## 1 Introduction

Globally, gastric cancer ranks fifth in incidence and second in cancer-related mortality, causing more than 800,000 deaths each year [1], 2]. The burden is highest in Eastern Asia, Eastern Europe, and South America [3], 4]. Locally advanced gastric cancer (LAGC) carries a particularly poor prognosis because of frequent recurrence and metastasis [5]. Neoadjuvant chemotherapy (NAC) has become a cornerstone of LAGC management [6]. In resectable disease, the FLOT regimen (5-fluorouracil, leucovorin, oxaliplatin, and docetaxel) achieves higher response rates and superior survival than older ECF/ECX combinations [7]. Across studies, NAC improves overall survival (OS) and disease-free survival (DFS) and may reduce postoperative morbidity [5], yet responses vary widely among patients [6]. This heterogeneity underscores the need for individualized treatment strategies and supports integrating molecularly targeted therapies and immunotherapy to enhance efficacy [7].

Accurate predictors could significantly enhance surgical outcomes by allowing for tailored surgical approaches [8], 9]. In parallel, advances in non-invasive gastric evaluation, such as magnetic-controlled capsule endoscopy incorporating the Kyoto classification, may facilitate earlier detection and structured surveillance, complementing biomarker-guided triage [10]. Inflammatory markers including the neutrophil-to-lymphocyte ratio (NLR), C-reactive protein (CRP), and albumin, together with tumor markers such as carcinoembryonic antigen (CEA) and CA19-9, have demonstrated prognostic value. Composite inflammation-based scores, notably the systemic inflammation response index (SIRI) and the cancer-inflammation prognostic index (CIPI), predict overall survival (OS) and disease-free survival (DFS) in colorectal cancer [11], 12]. Additional indices, including the inflammatory burden index (IBI), have been proposed to further improve predictive accuracy [12], 13]. Leveraging these combined indices can refine risk stratification and help anticipate therapeutic responses, facilitating more effective, individualized treatment planning [9], 14].

In this study, we evaluated whether dynamic changes in the combined inflammation–tumor marker index (CITI) during neoadjuvant chemotherapy (NAC) predict pathological response and thereby identify candidates for organ preservation. We also examined the prognostic value of CITI for disease-free survival (DFS) and overall survival (OS), and compared its predictive accuracy with that of individual markers used in routine practice.

## 2 Methods

### 2.1 Study design and setting

We conducted a prospective observational cohort study at Ningxia Medical University General Hospital from January 2018 through December 2023. The primary objective was to determine whether the combined inflammation–tumor marker index (CITI) measured during neoadjuvant chemotherapy (NAC) predicts pathological response and can help identify candidates for organ preservation.

**Informed consent:** Informed consent has been obtained from all individuals included in this study.

**Ethical approval:** The research related to human use has been complied with all the relevant national regulations, institutional policies and in accordance with the tenets of the Helsinki Declaration, and has been approved by the Ethics Committee of Ningxia Medical University General Hospital

### 2.2 Patient selection

#### 2.2.1 Inclusion criteria

1. Adults (≥18 years); 2. Histologically confirmed gastric adenocarcinoma; 3. Clinical stage cT2–T4 and/or node-positive disease (cN+) based on contrast-enhanced computed tomography (CT) of the abdomen and pelvis; 4. No radiographic evidence of distant metastasis (M0); 5. Eligible for neoadjuvant chemotherapy according to institutional standards; 6. Eastern Cooperative Oncology Group (ECOG) performance status 0–2; 7. Adequate organ function: hematologic (neutrophils ≥ 1.5 × 10^9^/L; platelets ≥ 100 × 10^9^/L), hepatic (total bilirubin < 1.5 × upper limit of normal [ULN]; ALT and AST <2.5 × ULN), and renal (creatinine clearance ≥ 50 mL/min); 8. Willing and able to comply with the protocol, including scheduled follow-up.

#### 2.2.2 Exclusion criteria

1), evidence of distant metastases or peritoneal carcinomatosis; 2), Prior systemic chemotherapy or radiotherapy for gastric cancer; 3), An active second primary malignancy within the past 5 years; 4), uncontrolled comorbidities that would preclude safe chemotherapy or surgical intervention; 5), inability or unwillingness to provide informed consent.

### 2.3 Neoadjuvant chemotherapy protocol

All enrolled patients received neoadjuvant chemotherapy (NAC) according to institutional protocols. Two primary regimens were used:

#### 2.3.1 FLOT

5-fluorouracil (5-FU) 2,600 mg/m^2^ as a 24-h continuous infusion, leucovorin 200 mg/m^2^, oxaliplatin 85 mg/m^2^, and docetaxel 50 mg/m^2^, all on day one; cycles were repeated every 2 weeks for 4–6 cycles, at the treating physician’s discretion and according to tolerance.

#### 2.3.2 SOX

Oral S-1 (weight-based dose) on days 1–14 plus oxaliplatin 130 mg/m^2^ intravenously on day 1; cycles were repeated every 3 weeks for 4–6 cycles.

#### 2.3.3 Dose modifications and assessments

Dose delays and reductions were permitted for toxicity – particularly hematologic adverse events and peripheral neuropathy – and were recorded prospectively. During NAC, evaluations included physical examination, complete blood count and serum chemistry panels, and adverse-event grading per CTCAE v5.0.

#### 2.3.4 Restaging and surgery

After completion of NAC, patients underwent restaging with computed tomography (CT) of the chest, abdomen, and pelvis. Curative-intent surgery was typically scheduled 3–6 weeks after the final chemotherapy cycle unless there was evidence of disease progression or the patient was deemed unfit for surgery.

### 2.4 Combined inflammation–tumor marker index (CITI)

The combined inflammation–tumor marker index (CITI) was constructed as a composite integrating inflammatory markers – C-reactive protein (CRP), albumin (ALB), and the neutrophil-to-lymphocyte ratio (NLR) – and tumor markers – carcinoembryonic antigen (CEA) and carbohydrate antigen 19-9 (CA19-9).

Peripheral blood was collected at three prespecified time points: baseline (T0, within 1 week before initiating neoadjuvant chemotherapy [NAC]); mid-NAC (T1, at cycle 2 or 3, depending on regimen); and post-NAC (T2, within 2 weeks after completion of the last NAC cycle and before surgery). For each timepoint, CRP (mg/L), albumin (g/L), CEA (ng/mL), and CA19-9 (U/mL) were natural-log transformed to reduce right skew and then standardized to z-scores, and NLR was standardized directly (unitless). Each marker was combined to yield a CITI score. We derived weights using an independent retrospective pilot cohort to predict major pathologic response (MPR; Becker 1a–1b) after NAC. After the same preprocessing, we fit a multivariable logistic regression with predictors *Z*_NLR_, *Z*_CRP_, *Z*_Albumin_, *Z*_CEA_, *Z*_CA19-9_. To guard against overfitting, coefficients were uniformly shrunk using bootstrap-estimated calibration slope. Final weights were the shrunk coefficients rescaled to a convenient magnitude (albumin retained a negative sign). Stability and performance were assessed via bootstrap resampling (1,000 iterations) to estimate optimism-corrected discrimination (AUC) and calibration (intercept/slope).

Dynamic changes in CITI were classified as significant decline (decrease ≥ 1 point), stable (±<0.5 points), or significant rise (increase ≥ 1 point).

CITI score was calculated by the following formula:

$$\text{CITI}=0.25\cdot \left(Z_{NLR}\right)+0.30\cdot \left(Z_{CRP}\right)-0.20\cdot \left(Z_{Albumin}\right)+0.25\cdot \left(Z_{CEA}\right)+0.40\cdot \left(Z_{CA19-9}\right)$$

where:

*Z*_NLR_ is the standardized (*z*-scored) neutrophil-to-lymphocyte ratio (unitless).

*Z*_CRP_ is the standardized C-reactive protein concentration (mg/L).

*Z*_Albumin_​ is the standardized albumin level (g/L).

*Z*_CEA_ is the standardized carcinoembryonic antigen level (ng/mL).

*Z*_CA19−9_ is the standardized carbohydrate antigen 19-9 level (U/mL).

*w*_1_ to *w*_5_ are the weighting coefficients.

Each biomarker is first log-transformed or z-score normalized to mitigate skew (common with tumor markers) and to ensure all variables contribute comparably.

### 2.5 Surgery and pathologic assessment

All patients were initially scheduled for curative-intent resection – total or subtotal gastrectomy with *D*2 lymphadenectomy. The choice between total and subtotal gastrectomy was determined by tumor location, extent, and the surgeon’s discretion. For patients who did not proceed to surgery, the reason – disease progression, patient preference, or medical inoperability – was recorded prospectively.

*Organ Preservation Criteria*: All patients were planned for curative resection (total or subtotal gastrectomy with *D*2 lymphadenectomy). Post-NAC decisions were made in a multidisciplinary team including a surgical oncologist, medical oncologist, gastroenterologist, diagnostic radiologist, and GI pathologist. Candidates were considered only when all of the following were present after NAC:

(a) Radiology: on contrast-enhanced CT, no new lesions, no radiologic progression, no suspicious nodes; gastric wall thickening consistent with post-treatment change was permissible.
(b) Endoscopy: regression to a flat scar or minimal residual lesion without contact bleeding; targeted biopsies negative or showing only minute residual atypia without definite invasive carcinoma.
(c) Multidisciplinary concordance: imaging and endoscopic/biopsy findings consistent with clinical complete or near-complete response, and tumor location judged technically amenable to endoscopic therapy or function-preserving resection.
(d) Supportive biomarker signal: ΔCITI ≥ 1.0 from baseline to post-NAC served as supportive evidence but was not used as a sole determinant; discordant CITI dynamics led to intensified assessment rather than automatic exclusion.

If findings were discordant, patients were not offered non-operative watch-and-wait. The default recommendation was standard gastrectomy. Rarely, if all anatomic criteria were favorable and uncertainty was limited to biomarkers alone, a limited/function-preserving resection could be considered after additional targeted biopsies and MDT consensus.

*Surveillance after organ-preserving management*: Every 3 months in year 1, then every 4–6 months in year 2, with high-definition endoscopy and targeted biopsies of the scar or any suspicious area. CT chest/abdomen/pelvis every 3–4 months in year 1, then every 6 months in year 2. CEA, CA19-9, CRP, albumin, and NLR were obtained at each visit. CITI was re-calculated to track dynamics during surveillance. Any biopsy-proven regrowth, new/enlarging suspicious nodes, or consistent rising CITI coupled with new radiologic/endoscopic suspicion prompted expedited salvage gastrectomy.

### 2.6 Sample size calculation

**Sample size and power.** We used a two-sample comparison of proportions to estimate the sample size. We hypothesized that patients with a favorable decline in CITI would have a pathologic complete response (pCR) rate of approximately 35 % versus 15 % in those without a favorable decline. With a two-sided *α* = 0.05 and 80 % power, the required sample size was ∼74 patients per subgroup. Allowing for ∼10–15 % dropout or non-evaluable cases, we targeted a total enrollment of 160–180 patients. Ultimately, 179 patients were recruited within the prespecified timeframe.

### 2.7 Statistical analysis

Patient demographics, treatments, and outcomes were stored in a secure electronic database. Continuous variables were summarized as mean ± standard deviation (SD) or median [interquartile range, IQR], as appropriate; categorical variables as counts (percentages). Changes in CITI across time (baseline, mid-NAC, post-NAC) were assessed with paired *t* tests or Wilcoxon signed-rank tests, as appropriate. Between-group differences in CITI for responders versus non-responders were tested with unpaired *t* tests (normally distributed) or Mann–Whitney *U* tests (non-normal). For multiple pathological-response categories (pCR, near-pCR, partial, minimal/no response), one-way ANOVA or Kruskal–Wallis tests were used with multiplicity-adjusted post-hoc comparisons. Disease-free survival (DFS) and overall survival (OS) were estimated by the Kaplan–Meier method from the date of surgery, and survival curves for favorable versus unfavorable CITI groups were compared using log-rank tests. Multivariable Cox proportional-hazards models adjusted for baseline stage, histology, and other relevant covariates; results are reported as hazard ratios (HRs) with 95 % confidence intervals (CIs). All tests were two-sided, with *α* = 0.05 considered statistically significant.

*Multiplicity and subgroup analyses*: Subgroup comparisons were pre-specified as exploratory and reported with nominal, unadjusted *p*-values. Given the number of contrasts, there is an increased risk of type I error (false positives). We therefore emphasize effect sizes and 95 % CIs and interpret subgroup findings as hypothesis-generating pending confirmation in larger, independent datasets.

Analyses were performed using *R* (version 4.4) statistical software. Logistic regression (major pathologic response), Kaplan–Meier estimation, log-rank testing, Cox proportional-hazards modeling, and PH-assumption checks were implemented using the packages (stats, survival, boot, survminer, ggplot2, dplyr, and broom).

## 3 Results

### 3.1 Patient enrollment and baseline characteristics

Between January 2018 and December 2023, 179 patients with cT2–T4 and/or cN+ gastric adenocarcinoma were enrolled. Table 1 summarizes baseline demographics and tumor characteristics. The mean age was 57.8 ± 9.3 years, and 67.0 % were male. Tumors were most commonly located proximally (33.5 %) or in the distal stomach (35.8 %). Most patients had clinical T3 disease (55.9 %), and 41.3 % presented with N2 lymph-node involvement. The median baseline CITI was 4.0 (IQR 3.2–5.2), indicating a moderate composite inflammatory–tumor marker burden at diagnosis.

**Table 1:** Patient enrollment and baseline characteristics.

Characteristic	Value
Age (years), mean ± SD	57.8 ± 9.3
Sex, n (%)	
Male	120 (67.0 %)
Female	59 (33.0 %)
BMI (kg/m^2^), median (IQR)	23.2 (21.0–25.5)
ECOG performance status, n (%)	
0	75 (41.9 %)
1	76 (42.5 %)
2	28 (15.6 %)
Comorbidities, n (%)	
Hypertension	45 (25.1 %)
Diabetes	33 (18.4 %)
Other	9 (5.0 %)
Tumor location, n (%)	
Proximal	60 (33.5 %)
Middle	55 (30.7 %)
Distal	64 (35.8 %)
Clinical T stage, n (%)	
T2	20 (11.2 %)
T3	100 (55.9 %)
T4	59 (33.0 %)
Clinical N stage, n (%)	
N0	15 (8.4 %)
N1	52 (29.1 %)
N2	74 (41.3 %)
N3	38 (21.2 %)
Histological type, n (%)	
Intestinal	95 (53.1 %)
Diffuse	64 (35.8 %)
Mixed/other	20 (11.2 %)
Baseline laboratory values, median (IQR)	
CEA (ng/mL)	3.0 (1.9–5.2)
CA19-9 (U/mL)	42 (18–110)
CRP (mg/L)	6.2 (2.4–10.8)
Albumin (g/L)	38 (34–42)
Neutrophil-to-lymphocyte ratio (NLR)	2.4 (1.9–3.6)
Baseline combined inflammation-tumor marker index (CITI), mean ± SD	4.3 ± 1.7

### 3.2 Treatment delivery and compliance

All patients received neoadjuvant chemotherapy with either FLOT (5-fluorouracil, leucovorin, oxaliplatin, docetaxel) or SOX (S-1, oxaliplatin) (Table 2). Overall, 88.3 % completed the planned number of cycles. The most common grade 3–4 adverse events were neutropenia (23.5 %) and nausea/vomiting (11.2 %). Dose reductions were required in 17.9 % of patients, and 15.6 % experienced treatment delays longer than seven days. Eight patients (4.5 %) discontinued NAC owing to disease progression, and 10 (5.6 %) discontinued because of unacceptable toxicity.

**Table 2:** Treatment delivery, compliance, and adverse events.

Parameter	Value
Chemotherapy regimen, n (%)	
FLOT (5-FU, Leucovorin, Oxaliplatin, Docetaxel)	120 (67.0 %)
SOX (S-1, Oxaliplatin)	59 (33.0 %)
Median number of planned cycles (IQR)	4 (4–5)
Completed planned NAC, n (%)	158 (88.3 %)
Dose reductions, n (%)	32 (17.9 %)
Treatment delays > 7 days, n (%)	28 (15.6 %)
Reasons for NAC discontinuation	
Disease progression	8 (4.5 %)
Unacceptable toxicity	10 (5.6 %)
Patient withdrawal	3 (1.7 %)
Adverse events, n (% of total)	
Neutropenia (G3–G4)	42 (23.5 %)
Anemia (G3–G4)	18 (10.1 %)
Thrombocytopenia (G3–G4)	12 (6.7 %)
Nausea/vomiting (G3–G4)	20 (11.2 %)
Diarrhea (G3–G4)	7 (3.9 %)
Neuropathy (any grade)	35 (19.6 %)
Infections (any grade)	23 (12.8 %)
Hand-foot syndrome (G2–G3)	9 (5.0 %)
Fatigue (G2–G3)	21 (11.7 %)

### 3.3 CITI patterns during neoadjuvant chemotherapy

As summarized in Table 3, the median CITI declined from 4.0 (IQR 3.2–5.2) at baseline to 3.1 (IQR 2.4–4.1) post-NAC. Overall, 53.1 % of patients experienced a ≥1-point decrease in CITI from baseline to post-NAC, whereas 17.9 % showed a net increase. Patients with a ≥1-point decline were more likely to complete all planned chemotherapy cycles without major dose modifications. Conversely, those with stable or rising CITI more often had poorer treatment tolerance or disease progression during NAC.

**Table 3:** Combined inflammation–tumor marker index (CITI) patterns.

Parameter	Value
Baseline CITI (Pre-NAC)	
Mean ± SD	4.3 ± 1.7
Median (IQR)	4.0 (3.2–5.2)
CITI at mid-NAC	
Mean ± SD	3.7 ± 1.6
Median (IQR)	3.6 (2.8–4.8)
CITI post-NAC (pre-operative)	
Mean ± SD	3.2 ± 1.5
Median (IQR)	3.1 (2.4–4.1)
Change in CITI from baseline to mid-NAC	
Decreased ≥ 1 point, n (%)	68 (38.0 %)
Stable (±<0.5), n (%)	70 (39.1 %)
Increased ≥ 1 point, n (%)	41 (22.9 %)
Change in CITI from baseline to post-NAC	
Decreased ≥ 1 point, n (%)	95 (53.1 %)
Stable (±<0.5), n (%)	52 (29.1 %)
Increased ≥ 1 point, n (%)	32 (17.9 %)
Change in CITI from mid-NAC to post-NAC	
Decreased ≥ 1 point, n (%)	60 (33.5 %)
Stable (±<0.5), n (%)	92 (51.4 %)
Increased ≥ 1 point, n (%)	27 (15.1 %)

### 3.4 Pathologic response and surgical outcomes

Following NAC, 164 of 179 patients (91.6 %) underwent surgical resection (Table 4). Total gastrectomy was performed in 58.3 % of cases, and 36.8 % underwent subtotal gastrectomy. An R0 resection (negative margins) was achieved in 90.8 % of surgical patients. A pathologic complete response (pCR) occurred in 16.6 %, and an additional 9.2 % had near-pCR. The median number of lymph nodes examined was 24 per specimen.

**Table 4:** Pathological response and surgical outcomes.

Parameter	Value
Patients undergoing surgery, n (%)	164 (91.6 %)
Reasons for no surgery, n (%)	
Disease progression	7 (3.9 %)
Patient refusal	5 (2.8 %)
Medical inoperability	3 (1.7 %)
Type of surgery performed, n (%)	
Total gastrectomy	95 (58.3 %)
Subtotal gastrectomy	60 (36.8 %)
Other/extended resection	8 (4.9 %)
Resection margin status	
R0 (clear margins)	148 (90.8 %)
R1 (microscopic residual)	12 (7.4 %)
R2 (macroscopic residual)	3 (1.8 %)
Median number of lymph nodes retrieved (IQR)	24 (18–30)
Pathologic response	
pCR (complete response), n (%)	27 (16.6 %)
Near pCR (mandard 1b/becker 1b), n (%)	15 (9.2 %)
Partial response (mandard 2–3/becker 2), n (%)	78 (47.9 %)
Minimal/no response (mandard 4–5/becker 3), n (%)	43 (26.3 %)

### 3.5 Organ-preservation considerations

A subset of 15/179 (8.4 %) met MDT criteria for potential organ preservation, 11/15 (73.3 %) ultimately underwent an organ-preserving approach (endoscopic resection, function-preserving gastrectomy, or non-operative surveillance) (Table 5). During a median 12-month follow-up (range 6–24), 2/11 (18.2 %) experienced local regrowth and were successfully salvaged surgically (Table 6). Given the small size of this subgroup and relatively short follow-up, these outcomes are presented as descriptive and should be interpreted cautiously; standard gastrectomy remains the default approach, with organ-preservation reserved for rigorously vetted cases under close surveillance.

**Table 5:** Comparison of CITI and key outcomes in organ preservation versus standard surgery.

Parameter	Organ preservation candidates (*n* = 15)	Standard resection group (*n* = 164)	*p*-value
Baseline CITI (mean ± SD)	4.6 ± 1.5	4.2 ± 1.7	0.20
Post-NAC CITI (mean ± SD)	2.8 ± 1.3	3.4 ± 1.6	0.03
ΔCITI (baseline → post) (mean ± SD)	−1.8 ± 1.2	−0.8 ± 1.0	<0.01
Pathologic complete or near pCR, n (%)	6 (40.0 %)	36 (22.0 %)	0.04

**Table 6:** Organ-preservation considerations.

Parameter	Value
Patients meeting potential organ-preservation criteria, n (%)	15 (8.4 %)
Criteria for organ preservation	– Endoscopic and radiologic findings suggestive of complete regression – Negative or minimal residual disease on biopsy – Patient preference/consent
Organ-preservation approach	
Non-surgical “watch and wait”	5 (33.3 %)
Local excision/endoscopic resection	6 (40.0 %)
Partial/function-preserving gastrectomy	4 (26.7 %)
Patients actually undergoing organ-preserving strategy, n (%)	11 (6.1 % of total; 73.3 % of the 15 candidates)
Reasons for not pursuing organ preservation (n = 4 of 15)	
Patient preference for standard surgery	2 (50.0 %)
Uncertain biopsy results	1 (25.0 %)
Evolving nodal disease on imaging	1 (25.0 %)
Median follow-up after organ preservation (months)	12 (range 6–24)
Local recurrence among organ-preserved patients, n (%)	2 (18.2 %)
Time to local recurrence (months)	7 and 10
Treatment of local recurrence (salvage surgery)	2 (100 %)
Current status of organ-preserved patients	
No evidence of disease	9 (81.8 %)
Alive with disease	2 (18.2 %)
Deaths	0

Although baseline CITI was not significantly different among eventual responders versus non-responders (*p* = 0.20), the change in CITI from baseline to post-NAC was strongly correlated with pathologic outcomes (Table 6). Patients who achieved a pCR or near pCR demonstrated a mean CITI drop of −1.7 ± 1.2, compared to −0.7 ± 1.0 among those with partial or minimal/no response (*p* < 0.01). On multivariate logistic regression, a ≥1-point decrease in CITI emerged as an independent predictor for major pathologic response (OR = 2.76, 95 % CI 1.40–5.43; *p* = 0.02).

### 3.6 Survival outcomes

Table 7 illustrates OS and DFS for the entire cohort and stratified by favorable (≥1-point drop) versus unfavorable CITI changes. At a median follow-up of 24 months, the 3-year OS was 66.8 % for those with favorable CITI versus 52.7 % for unfavorable CITI (*p* = 0.04). Similarly, the 3-year DFS was 56.3 % vs 41.0 % (*p* = 0.06), respectively. A Cox proportional hazards model adjusted for baseline stage and histology confirmed that a significant decrease in CITI was associated with improved OS (HR = 0.60, 95 % CI 0.40–0.90; *p* = 0.01). In multivariable Cox models further adjusting for chemotherapy regimen (FLOT versus SOX) and baseline CITI, the association between a ≥1-point *Δ*CITI decline and overall survival remained independent (adjusted HR = 0.62, 95 % CI 0.43–0.91; *p* = 0.015), while disease-free survival showed a consistent, directionally favorable pattern (adjusted HR = 0.76, 95 % CI 0.55–1.06; *p* = 0.10). Baseline CITI was itself associated with outcomes (per-unit HR for OS = 1.12, 95 % CI 1.03–1.22; *p* = 0.008; per-unit HR for DFS = 1.09, 95 % CI 1.01–1.18; *p* = 0.028), and regimen did not materially alter inference (Supplementary Table S1).

**Table 7:** Survival outcomes.

Survival metric	Entire cohort (N = 179)	Favorable CITI (n = 95)	Unfavorable CITI (n = 84)	p-value
Median follow-up, months (range)	24 (6–48)	26 (8–48)	23 (6–45)	–
Overall survival (OS)				
Median OS, months (95 % CI)	48.0 (32.5–NR)	NR (40.0–NR)	42.0 (34.0–NR)	0.02
1-year OS, %	88.2 %	92.6 %	83.3 %	0.03
3-year OS, %	60.5 %	66.8 %	52.7 %	0.04
Disease-free survival (DFS)				
Median DFS, months (95 % CI)	34.0 (22.0–NR)	40.5 (28.0–NR)	25.5 (16.0–40.0)	0.01
1-year DFS, %	78.8 %	84.5 %	71.2 %	0.04
3-year DFS, %	49.2 %	56.3 %	41.0 %	0.06

### 3.7 Additional subgroup analyses

As summarized in Table 8, associations between CITI, NAC response, and outcomes varied by tumor location, histologic subtype, and regimen. Intestinal-type tumors had higher rates of major pathologic response (pCR + near-pCR) than diffuse-type tumors (31.6 % versus 15.6 %). Patients treated with FLOT exhibited a numerically higher pCR rate than those receiving SOX (18.3 % versus 8.5 %). Importantly, a baseline CITI < 4.0 was associated with a greater likelihood of achieving pCR or near-pCR (32.5 % versus 18.2 % for CITI ≥ 4.0) and with longer disease-free survival (DFS; log-rank *p* < 0.05). These findings highlight heterogeneity in treatment response across clinical subgroups and support individualized therapeutic strategies.

**Table 8:** Additional subgroup analyses.

Subgroup	pCR rate, n (%)	Major response (pCR + near pCR), n (%)	Favorable CITI, n (%)	Median DFS (months, 95 % CI)	Median OS (months, 95 % CI)	p-value
Tumor location						
Proximal (≥cardia) (n = 69)	9 (15.0)	15 (25.0)	30 (50.0)	32.0 (20.0–NR)	46.0 (28.0–NR)	0.05
Middle/distal (n = 119)	18 (15.1)	27 (22.7)	65 (54.6)	38.0 (25.0–NR)	48.0 (33.0–NR)	0.08
Histological Subtype						
Intestinal (n = 95)	20 (21.1)	30 (31.6)	58 (61.1)	40.5 (28.0–NR)	NR (40.0–NR)	0.01
Diffuse (n = 64)	5 (7.8)	10 (15.6)	20 (31.3)	25.0 (16.0–50.0)	38.0 (24.0–NR)	0.03
Mixed/other (n = 20)	2 (10.0)	3 (15.0)	8 (40.0)	22.0 (10.0–NR)	32.0 (18.0–NR)	0.10
Chemotherapy Regimen						
FLOT (n = 120)	22 (18.3)	32 (26.7)	70 (58.3)	35.0 (25.0–NR)	48.0 (32.0–NR)	0.02
SOX (n = 59)	5 (8.5)	10 (16.9)	25 (42.4)	30.0 (20.0–45.0)	40.0 (25.0–NR)	0.09
Baseline CITI						
Low (<4.0) (n = 80)	16 (20.0)	26 (32.5)	58 (72.5)	40.0 (28.0–NR)	NR (40.0–NR)	0.01
High (≥4.0) (n = 99)	11 (11.1)	18 (18.2)	37 (37.4)	28.0 (18.0–50.0)	44.0 (27.0–NR)	0.05

Subgroup analyses are exploratory; p-values are nominal (unadjusted for multiple comparisons) and should be interpreted cautiously due to potential type I error inflation. Effect sizes and consistency across related endpoints should guide inference rather than p-values alone.

## 4 Discussion

Our findings show that a substantial proportion of patients achieved a pathological complete response (pCR), and that greater declines in the combined inflammation–tumor marker index (CITI) were associated with markedly improved survival. Patients with the largest CITI reductions were more likely to attain pCR and experienced longer disease-free survival (DFS) than those with stable or rising CITI. These results indicate that serial CITI monitoring can predict response to neoadjuvant chemotherapy and help identify candidates for organ preservation.

Our findings suggest that dynamic changes in the combined inflammation–tumor marker index (CITI) capture both tumor burden and the host inflammatory milieu, consistent with mechanistic links proposed in prior work on inflammatory and tumor biomarkers [15], [16], [17]. In our cohort, higher CRP, NLR, and CA19-9 levels were associated with greater chemoresistance, aligning with the concept that systemic inflammation promotes tumor proliferation and immune evasion [18], [19], [20]. Conversely, a post-chemotherapy decrease in CITI was associated with enhanced chemosensitivity, plausibly reflecting attenuation of tumor-promoting inflammation and improved systemic control [16], 21]. These observations accord with studies in colorectal and gastric cancers showing that decreases in CRP and NLR predict better responses to chemotherapy [15], 22], 23].

Clinically, a marked decline in CITI helped identify a subset of patients who could safely undergo organ-preserving management – either limited gastric resection or a “watch-and-wait” surveillance strategy. Among the 15 patients who met prespecified organ-preservation criteria, 11 elected function-preserving management; two developed local recurrences, both of which were successfully treated with salvage surgery. These short-term outcomes compare favorably to those in standard resection patients, suggesting that biomarker-driven selection may help identify candidates warranting further evaluation in larger trials [24]. Similar principles have been applied in esophageal and rectal cancers, where endoscopic techniques and careful patient monitoring have allowed non-operative strategies in well-selected cases [25], [26], [27], [28], [29], [30], [31].

Our findings support that combining inflammatory markers (CRP, NLR, albumin) with tumor markers (CEA, CA19-9) provides a more comprehensive predictive framework than single markers in gastric cancer, consistent with prior studies [32], [33], [34], [35]. In particular, composite scores such as the NLR–CA19-9 index and related integrative metrics have demonstrated improved prognostic discrimination and greater utility for therapeutic decision-making than conventional single-marker strategies [33], [34], [35]. In line with the concept that systemic inflammation modulates tumor biology, reductions in inflammatory burden correlate with improved outcomes and enhanced chemosensitivity [15], [16], [17]. By leveraging multimarker information that reflects both tumor burden and host immune status, clinicians may more accurately stratify patients for therapy intensification or, conversely, consider organ preservation according to the individual CITI trajectory.

Although biomarker-driven models have advanced in gastric cancer, organ preservation remains comparatively underexplored relative to rectal cancer, where watch-and-wait strategies are well established [36], [37], [38], [39], [40]. Nevertheless, experience from other gastrointestinal (GI) malignancies – notably esophageal cancer, in which endoscopic resection and immunotherapy are used in selected cases – suggests that rigorous eligibility criteria and structured surveillance can enable non-operative management in highly responsive patients [25], [26], [27], [28], [29], [30], [31]. In our cohort, substantial post-NAC declines in CITI may help identify a subset of patients amenable to organ-preserving strategies. As in rectal cancer, however, successful implementation hinges on robust patient selection and timely salvage interventions to limit the risk of local regrowth while maintaining favorable survival [41], [42], [43], [44], [45].

Compared with established inflammation-based composites, CITI adds two features that are directly relevant to predicting NAC response in gastric cancer. It is dual-domain and explicitly dynamic, using on-treatment change rather than a single baseline snapshot. Prior work shows that inflammation-centric indices can carry response signal associates with NAC sensitivity and prognosis in locally advanced gastric cancer, and dynamic SII tracks with higher TRG 0/1 rates and better survival after NAC [46], 47]. Ratios that blend inflammation and nutrition or tumor burden also help distinguish pathological responders to NAC [48]. By contrast, the modified Glasgow Prognostic Score is strongly prognostic in perioperative FLOT cohorts but is not primarily designed as a treatment-response readout [49]. In our cohort, a ≥1-point ΔCITI decline was associated with higher pCR and improved OS, suggesting that a dynamic, dual-domain composite may capture chemosensitivity more directly than baseline inflammation-only scores while remaining simple to operationalize alongside imaging and biopsy in MDT review. Head-to-head, prospective comparisons of ΔCITI versus SII/SIRI, CAR, and mGPS for predicting NAC response are warranted to confirm incremental value.

Compared with rectal and esophageal models, progress toward organ preservation in gastric cancer has lagged for several reasons. Post-NAC restaging is less reliable in the stomach: CT/EUS/targeted biopsies often struggle to distinguish fibrosis from residual tumor, making a reproducible clinical complete response difficult to define and increasing the risk of undertreatment [50]. The successes in other GI sites rely on specific preconditions, notably chemoradiation and rigorous response-assessment frameworks. In rectal cancer, watch-and-wait strategies within total neoadjuvant therapy achieve durable organ preservation in a substantial proportion of patients under strict surveillance [51]. In esophageal cancer, the preSANO program’s multimodal evaluation (bite-on-bite biopsies, EUS-FNA, and imaging) improves detection of residual disease and underpins active-surveillance protocols [52]. Treatment paradigms also differ. Contemporary gastric pathways are predominantly chemotherapy-based (e.g., FLOT), where complete-response rates remain modest compared with chemoradiation settings [53]. Early and occult nodal involvement in gastric cancer heightens concern about oncologic safety and salvageability when relying on local assessments alone. Reflecting these constraints, in our cohort only 15 out of 179 met MDT criteria for potential organ preservation, 11 of them ultimately underwent organ-preserving management, and 2 out of 11 experienced local regrowth, both successfully salvaged under a predefined protocol. Accordingly, we positioned ΔCITI ≥ 1 as supportive rather than determinative evidence, required concordant imaging plus endoscopy/biopsy, and mandated tight surveillance with prespecified salvage triggers. Taken together, these data support selective, cautious use of organ-preserving strategies in gastric cancer and underscore the need for longer follow-up and multicenter standardization.

This study has limitations. First, it was a single-center cohort with a modest sample size, which limits statistical power and may reduce generalizability. Second, although the trial was prospectively designed, the CITI weighting scheme and cut-off were derived from retrospective pilot data, introducing a risk of overfitting; these components require external validation and pre-specification in future studies. Third, treatment regimens, surgical approaches, and organ-preservation decisions were determined by a multidisciplinary team without randomization, raising the possibility of confounding by indication and selection bias that multivariable adjustment may not fully address. Additionally, the organ-preservation results in our series are encouraging, but they are based on a small, highly selected subgroup with limited follow-up, and thus are hypothesis-generating rather than definitive. These caveats warrant cautious interpretation of the findings and underscore the need for larger, multi-center studies with longer follow-up and pre-specified statistical plans to confirm the prognostic utility of dynamic CITI changes.

Despite these limitations, our data indicate that dynamic reductions in the combined inflammation–tumor marker index (CITI) during neoadjuvant chemotherapy are associated with a higher likelihood of pathological complete response (pCR), favorable survival, and successful organ-preserving management in locally advanced gastric cancer. CITI is a readily obtainable, cost-effective biomarker that integrates host inflammatory status with tumor burden and may assist multidisciplinary teams in tailoring treatment intensity and resection strategies. Prospective, multicenter studies with prespecified cut-offs and extended follow-up are warranted to validate these findings and to develop CITI-guided algorithms for individualized surgical decision-making.

## Supplementary Material

Supplementary Material
