# Supplementary material for: Dynamic variation in a combined inflammation–tumor marker index during neoadjuvant chemotherapy and its value for organ-preservation decisions in gastric cancer
Source: Open Life Sci. 2026 Jan 23;21(1):20251238. doi: 10.1515/biol-2025-1238 (PMC12917559; doi:10.1515/biol-2025-1238)
Supplement: Supplementary file 1 — Supplementary Material [file j_biol-2025-1238_suppl_001.docx]

**Supplementary Table S1. Multivariable Cox models for OS and DFS**

| **Endpoint** | **Covariate** | **Category / Scale** | **Adjusted HR** | **95% CI** | **p‑value** |
| --- | --- | --- | --- | --- | --- |
| **Overall Survival (OS)** | **ΔCITI (favorable)** | ≥ 1‑point decline vs <1 | **0.62** | **0.43–0.91** | **0.015** |
|  | Regimen | **FLOT vs SOX** | 0.82 | 0.60–1.13 | 0.22 |
|  | Baseline CITI | per 1‑unit (continuous, centered) | **1.12** | **1.03–1.22** | **0.008** |
|  | Clinical T | **T3 vs T2** | 1.18 | 0.77–1.79 | 0.45 |
|  |  | **T4 vs T2** | **1.57** | **1.01–2.44** | **0.047** |
|  | Clinical N | **N1 vs N0** | 1.26 | 0.74–2.14 | 0.40 |
|  |  | **N2 vs N0** | 1.63 | 0.98–2.72 | 0.06 |
|  |  | **N3 vs N0** | **1.94** | **1.13–3.33** | **0.017** |
|  | Histology | **Diffuse vs Intestinal** | 1.28 | 0.90–1.83 | 0.17 |
|  |  | **Mixed vs Intestinal** | 1.15 | 0.67–1.98 | 0.62 |
| **Disease‑Free Survival (DFS)** | **ΔCITI (favorable)** | ≥ 1‑point decline vs <1 | **0.76** | **0.55–1.06** | **0.10** |
|  | Regimen | **FLOT vs SOX** | 0.89 | 0.65–1.22 | 0.47 |
|  | Baseline CITI | per 1‑unit (continuous, centered) | **1.09** | **1.01–1.18** | **0.028** |
|  | Clinical T | **T3 vs T2** | 1.25 | 0.86–1.82 | 0.24 |
|  |  | **T4 vs T2** | **1.65** | **1.12–2.44** | **0.012** |
|  | Clinical N | **N1 vs N0** | 1.34 | 0.81–2.22 | 0.25 |
|  |  | **N2 vs N0** | **1.72** | **1.06–2.80** | **0.027** |
|  |  | **N3 vs N0** | **2.02** | **1.22–3.36** | **0.006** |
|  | Histology | **Diffuse vs Intestinal** | 1.33 | 0.95–1.86 | 0.09 |
|  |  | **Mixed vs Intestinal** | 1.20 | 0.71–2.02 | 0.50 |
